# Temperature-related atypical first-bite syndrome: a rare case report

**DOI:** 10.3389/froh.2025.1678707

**Published:** 2025-12-11

**Authors:** Zuntai Li, Ke Zhang, Yifan Dong, Zihui Wang, Chunxia Zhang, Long Su, Bo Zhao

**Affiliations:** 1 Department of Stomatology, Tianjin First Central Hospital, Tianjin, China; 2 Key Laboratory of Advanced Intelligent Protective Equipment Technology (Hebei University of Technology), Ministry of Education, Tianjin, China; 3 NHC Key Laboratory of Critical Care Medicine, Tianjin First Central Hospital, Tianjin, China; 4 Department of Ophthalmology, The Second Hospital of Tianjin Medical University, Tianjin, China; 5 Institute of Orbital Disease, The Second Hospital of Tianjin Medical University, Tianjin, China

**Keywords:** first bite syndrome, temperature-related, Marcus-Gunn syndrome, treatment, case report

## Abstract

First bite syndrome (FBS) manifests is characterized by severe parotid pain triggered by the first bite of food, with or without muscle spasms. The pain typically diminishes with subsequent bites. We report a case of 32-year-old male with Marcus-Gunn syndrome (30-year history), no prior head or neck surgery, tumors, infections, or temporomandibular joint disease. Since age of 15, he experiences bilateral parotid pain and spasms exclusively upon consuming ice cream in high ambient temperatures post-summer exercise. Symptoms resolve spontaneously within 30 s and persist despite trials of heat therapy, massage, and physiotherapy. While approximately 42% of FBS cases have identifiable etiology, others implicate aberrant activity in the auriculotemporal, greater auricular, or cervical sympathetic nerves. In this patient, symptoms occur only with cold food ingestion in hot environments, suggesting involvement of the trigeminal nerve's mandibular branch in oral thermosensation. To our knowledge, this is the first reported case linking FBS symptoms to temperature sensation.

## 1 Introduction

First bite syndrome (FBS), first described by Netterville et al. (1998), is defined by severe, sharp, or spasmodic parotid pain upon the initial bite of food (1). Pain typically subsides within seconds. Netterville et al (2). proposed that FBS primarily localizes to the parotid gland.

The pathogenesis of first bite syndrome (FBS) remains incompletely understood (3). Approximately 42% of patients report a history of parotid surgery, facial malignancy, or cervical/prevertebral space infection, while the remainder present idiopathically (4). Current treatments like local physiotherapy and botulinum toxin type A injections show preliminary efficacy, but a systematic evaluation of their therapeutic outcomes in FBS is lacking (5).

This case report describes an atypical presentation of FBS. Pain upon the initial bite is triggered by food intake and environmental temperature, despite the absence of trauma, surgery, infection, or temporomandibular joint disease. To our knowledge, this is the first reported case of FBS with temperature-dependent triggers in an idiopathic setting.

## 2 Case description

A 32-year-old male presented to our dental clinic with intermittent mastication-triggered pain. He reported bilateral parotid pain upon the initial bite of cold foods since the age of 15, with each episode lasting 30–60 s before spontaneous resolution. The patient denied any history of trauma, familial genetic disorders, temporomandibular joint pathology, or neoplasms. His medical history was significant for Marcus-Gunn syndrome ince infancy (approximately 30 years). The publication of this case report, including all associated images, tables, and data, was conducted with the patient's full informed consent. Written authorization was obtained prior to submission, following institutional ethical guidelines.

## 3 Diagnostic assessment

### 3.1 Examination

**Figure 1:**
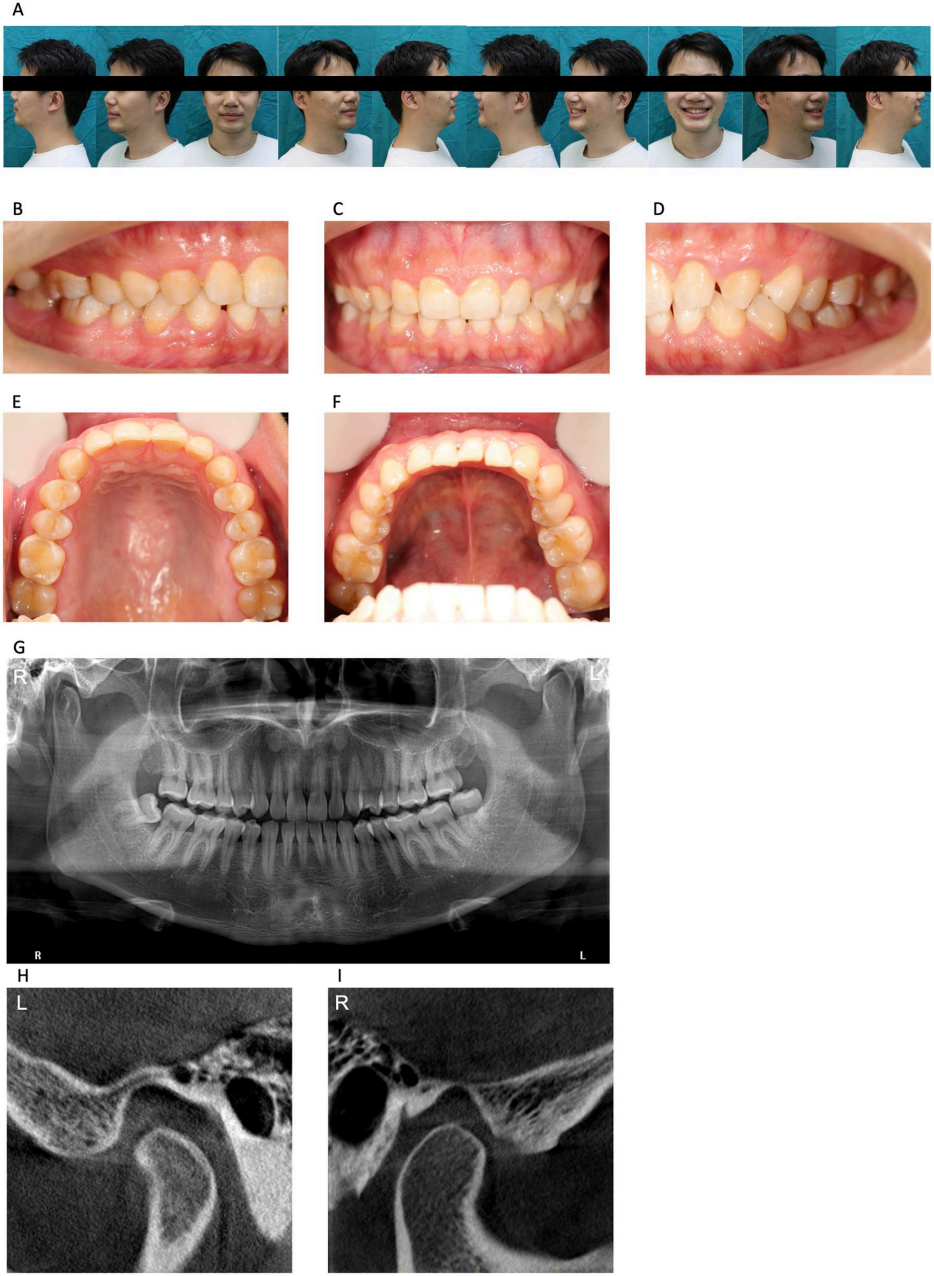
Clinical and imaging examinations of the patient. **(A)** Photos of the patient at rest and smiling in both anteroposterior and lateral positions; **(B)** The patient's right occlusal condition; **(C)** The patient is in a median occlusal state; **(D)** The patient's left occlusal condition; **(E)** Intraoral photograph of the patient's upper jaw; **(F)** A photo of the patient's mandibular opening; **(G)** Curved surface tomography imaging examination of the patient; **(H)** CBCT examination of the patient's left temporomandibular joint; **(I)** CBCT examination of the patient's right temporomandibular joint;.

Facial symmetry was unremarkable. Bilateral temporomandibular joint (TMJ) palpation revealed no tenderness. Maximum interincisal opening measured 38 mm with normal trajectory and absence of joint sounds (Figure 1). Cone-beam computed tomography (CBCT) of both TMJs showed symmetrical condyles without evidence of resorption or remodeling of the articular eminences (Figure 1), effectively ruling out structural joint pathology.

To exclude odontogenic pain, cone-beam computed tomography (CBCT) revealed no carious lesions except in the third molars (teeth28, 38, 48; Figure 1). Intraoral examination showed no tenderness to percussion and normal pulp sensibility (tested via cold, heat, and electric pulp testing) in all teeth (Figure 1).

To further confirm the diagnosis, with the patient's consent, we conducted a clenching test. After the patient gritted his teeth for 5 s, a significant local pain response occurred. We performed a comprehensive myofascial trigger point examination according to standard protocols. The superficial and deep portions of the masseter muscle were palpated extraorally using the index, middle, and ring fingers. The temporalis muscle was systematically evaluated bilaterally through digital palpation across its entire course. Intraoral palpation of the medial pterygoid muscle was conducted using the index finger directed toward the pterygomandibular region. The lateral pterygoid muscle function was assessed via palpation posterior and superior to the maxillary tuberosity. All examinations were performed under standardized pressure (approximately 2 lbs). None of the palpation maneuvers elicited the characteristic parotid pain reported by the patient. Based on this, we can initially rule out the similar disease of masseter myofascial pain.

**Figure 2:**
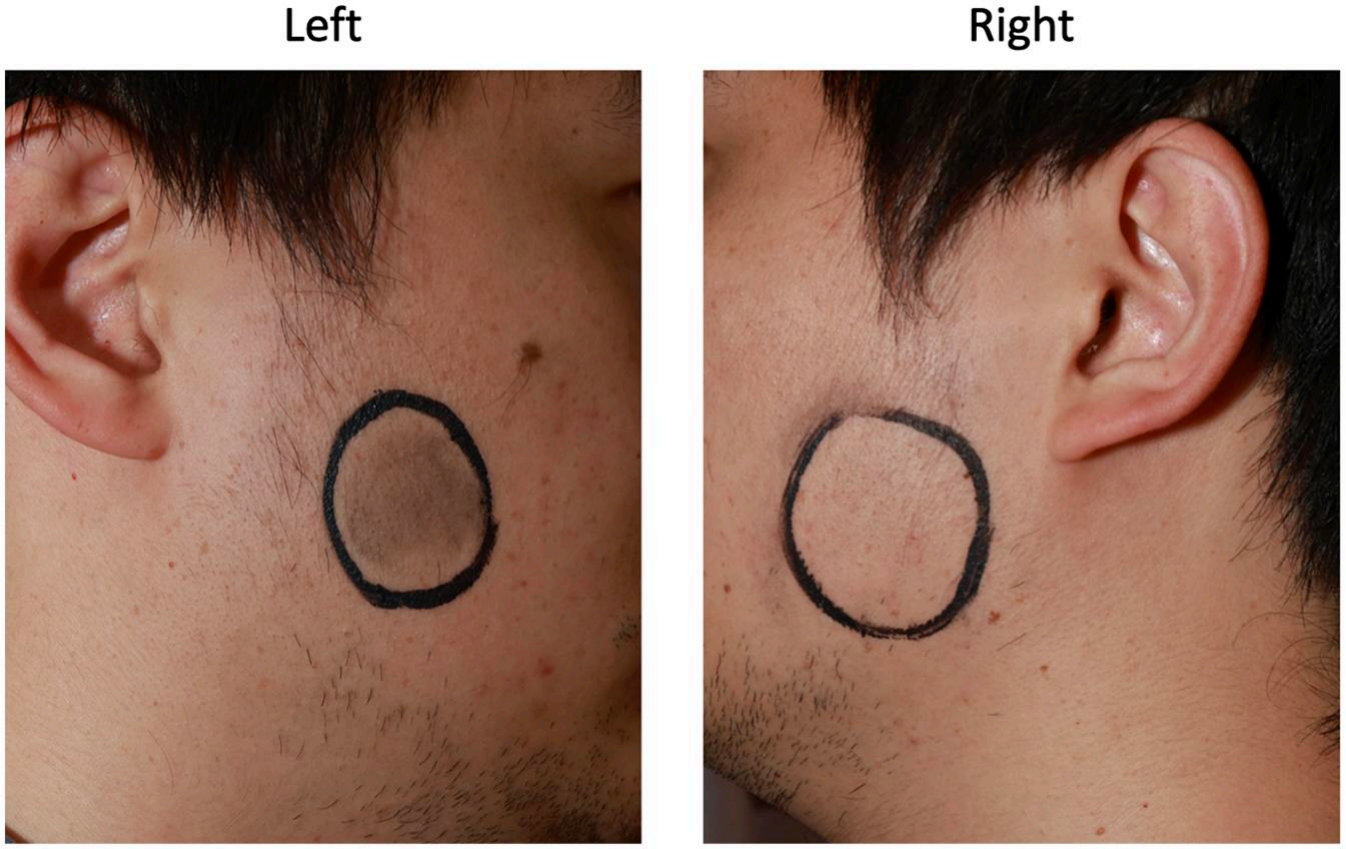
A facial map of the pain area drawn based on the patient's description of the pain area during an FBS attack.

Following informed consent, we clinically reproduced the pain symptoms under controlled conditions. Variables included ambient temperature (cold/hot), pre-test physical exertion status, and food temperature. Environmental conditions were standardized to hot (outdoor summer midday, 40 °C) and cool (air-conditioned indoor, 26 °C). Food stimuli comprised cream cake (20 °C), ice cream (−5 °C), and shaved ice (−20 °C). Exercise status was defined by 5-min aerobic jogging vs. 5-min seated rest. Pain was only reproducibly triggered when consuming cold food after exercise in a hot environment (Table 1). Using cosmetic mapping, we document the bilateral parotid pain distribution (Figure 2).

**Table 1:** The trigger conditions of FBS of this case.

Ambient temperature	Jogging/Rest	Food type	FBS occurs
Hot	Jogging	Comprised cream cake	No
		Ice cream	No
		Shaved ice	Yes
	Rest	Comprised cream cake	No
		Ice cream	No
		Shaved ice	No
Cool	Jogging	Comprised cream cake	No
		Ice cream	No
		Shaved ice	No
	Rest	Comprised cream cake	No
		Ice cream	No
		Shaved ice	No

We assessed patient pain level using the Wong-Baker FACES Pain Rating Scale and recorded pain duration. The baseline data (Table 2, Before Treatment) indicated an average pain level of 6.75 ± 0.97 and an average pain duration of 44.25 ± 11.91 s.

**Table 2:** The duration and degree of pain during FBS symptoms.

Before treatment				After treatment			
No.	onset time	Degree of pain	Duration(s)	No.	onset time	Degree of pain	Duration(s)
1	25/5/13 16:20	6	42	1	25/7/13 10:13	8	41
2	25/5/19 13:20	8	53	2	25/7/14 14:08	8	19
3	25/5/21 11:30	6	32	3	25/7/15 13:00	8	35
4	25/5/24 14:03	8	40	4	25/7/17 10:00	4	67
5	25/5/27 12:05	6	37	5	25/7/17 16:37	8	54
6	25/5/30 12:30	8	59	6	25/7/18 11:50	6	23
7	25/6/02 12:03	6	28	7	25/7/19 09:46	8	29
8	25/6/03 15:07	6	63	8	25/7/19 15:13	8	45
Mean ± SD		6.75 ± 0.97	44.25 ± 11.91	Mean ± SD		7.25 ± 1.39	39.13 ± 15.09

The Ophthalmology Department of the Second Affiliated Hospital of Tianjin Medical University provided assistance for the diagnosis of Marcus-Gunn syndrome in this patient. During the examination, the clinician observed that the patient only experienced rhythmic twitching of the left upper eyelid when chewing with the right teeth. This movement rhythm was the same as that of oral chewing, but this symptom did not occur when chewing on both sides. Collectively, these clinical features supported the diagnosis of Marcus-Gunn syndrome. Ophthalmic examination revealed a levator muscle strength of 13 mm in the right eye and 12 mm in the left. The static palpebral fissure height was symmetric, measuring 8 mm bilaterally. Dynamic evaluation, however, revealed a mild left-sided Marcus Gunn jaw-winking phenomenon, characterized by elevation of the left upper lid to 10 mm during mastication. Given the absence of functional impairment or significant ptosis, surgical intervention was not indicated, and periodic observation was recommended.

### 3.2 Treatment and prognosis

The patient was informed about the treatment options, including local physiotherapy and botulinum toxin injection. He opted to proceed with the physiotherapy plan. The patient underwent local electrical stimulation, massage and hot compress for one week in the hope of improving the local muscle tension state and the patient's FBS symptoms. And made an appointment for a follow-up visit four weeks.

During the four-week follow-up period, the patient received localized thermotherapy at least twice daily (≥20 min/session) and underwent electroacupuncture-based massage therapy administered by the Department of Integrated Traditional Chinese and Western Medicine (≥2 sessions/week, ≥30 min/session). Regular follow-up assessments were conducted per the treatment protocol.

During the follow-up visit four weeks later, we recorded the pain perception and duration of the patients during several episodes (Table 2 After Treatment) and analyzed them with the data before treatment, using the ANOVA method. The results showed that the degree of pain was 7.25 ± 1.39(mean ± SD), and the duration was 39.13 ± 15.09 s (mean ± SD). There was no significant difference in the pain duration and pain grade of the patients (*P* > 0.05), suggesting that local physiotherapy was ineffective.

To further alleviate the related symptoms, we recommend that the patient undergo botulinum toxin injection treatment. After fully understanding the advantages and complications of botulinum toxin, the patient adopted a plan of giving up cold food after intense exercise and refused the subsequent treatment.

## 4 Discussion

First bite syndrome (FBS) is characterized by parotid pain and muscle spasms during mastication (6). Its pathogenesis remains incompletely understood, with two prevailing hypotheses: Sympathetic Denervation Hypothesis (Netterville): Proposes FBS results from hypersensitivity following damage to parotid sympathetic fibers. This is supported by cases developing after carotid endarterectomy (7) and parotid infiltration by Hodgkin's lymphoma (8). Parasympathetic Dysregulation Hypothesis (Gardner): Suggests pain arises from unopposed parasympathetic activity causing vasodilation and sensory fiber stretching (9). This mechanism involves sympathetic hyperactivity or residual innervation after cervical ganglionectomy (9).

Our patient presented without surgical or neoplastic FBS triggers. However, he has a 30-year history of congenital Marcus-Gunn syndrome—caused by aberrant innervation between the trigeminal nerve's mandibular branch (masticatory muscles) and the oculomotor nerve (levator palpebrae superioris). This confirms embryonic neurodevelopmental abnormalities. Given the co-occurrence of FBS, we propose his symptoms represent primary FBS resulting from congenital neural dysregulation.

Marcus Gunn phenomenon is a congenital synkinetic ptosis resulting from aberrant innervation between the trigeminal and oculomotor nerves. It manifests as eyelid retraction upon jaw movement and accounts for 2%–13% of congenital ptosis cases (10). The classic “jaw-winking” phenomenon involves involuntary contraction of the ipsilateral levator palpebrae superioris muscle, synchronized with contraction of the lateral pterygoid muscle during jaw deviation. This sign is pathognomonic and establishes a definitive clinical diagnosis without requiring confirmatory imaging or serological testing (11). Characteristic manifestations of congenital Marcus Gunn syndrome can be observed as early as the neonatal period during feeding, typically persist through adolescence, and show no tendency to resolve in later life (12). In the present case, the patient exhibits obvious unilateral jaw movement with contralateral eyelid retraction since childhood. The symptoms show no significant improvement with age, which is consistent with the typical Marcus Gunn phenomenon and confirms the diagnosis of Marcus Gunn syndrome.

While Marcus Gunn syndrome has historically been classified as a genetic disorder, a 2024 case report has broadened its etiological spectrum to include post-traumatic origins (13). Current evidence indicates that the synkinesis between the lateral pterygoid and levator palpebrae superioris muscles results from aberrant connectivity between the mandibular branch of the trigeminal nerve (CN V) and the oculomotor nerve (CN III) (14). Intriguingly, this phenomenon has been speculated to reflect the persistence of a primitive reflex observed in amphibians such as Xenopus, which facilitates gaze stabilization during prey capture (15). Two primary pathophysiological hypotheses have been proposed: the first involves central nuclei crosstalk or peripheral misinnervation, where the anatomical proximity of the trigeminal motor nucleus (pons) to the oculomotor nucleus (midbrain) may permit aberrant neural signaling—impulses intended for jaw deviation inadvertently activating the levator palpebrae superioris subnucleus (16). During embryogenesis (weeks 5–8), a critical window for craniofacial innervation, mandibular nerve axons may aberrantly innervate the developing levator palpebrae superioris, thereby co-opting its function (17), a mechanism supported by intraoperative observations that trigeminal motor root stimulation elicits levator contraction in 20% of normal patients undergoing microvascular decompression (17). The second, termed the “release hypothesis,” posits that Marcus Gunn syndrome represents a phylogenetically suppressed reflex in mammals that can re-emerge under specific pathological conditions (14, 18). The present case is notably rare among Marcus Gunn syndromes (approximately 5.6%) (19), characterized by the absence of concurrent ptosis and a unique trigemino-oculomotor synkinesis between the left lateral pterygoid and levator palpebrae superioris muscles. This manifestation confirms abnormal development of masticatory muscles—particularly the extra-wing muscles—and their innervating nerves, aligning with the neurodevelopmental theory underlying First Bite Syndrome (FBS) etiology. However, due to the specific triggering conditions of FBS, the coexistence of both syndromes was not recognized until adolescence.

Differential diagnosis remains a critical component in the diagnostic workup of First Bite Syndrome (FBS) (1). Key conditions requiring distinction include facial neoplasms, medial pterygoid muscle hyperactivity, masseter myofascial pain, and radiation-induced osteonecrosis. In the present case, the absence of palpable masses and the prolonged clinical course effectively excluded malignant facial tumors, obviating the need for highly radioactive imaging modalities such as PET-CT. Medial pterygoid hyperactivity, a clinically overlapping entity, typically demonstrates load-dependent pain exacerbation and restricted mandibular movement (20). While the initial physical examination revealed no muscle tenderness or occlusal abnormalities, dynamic electromyography (EMG) during symptomatic episodes is necessary to definitively exclude masseter myofascial pain. The diagnosis of this condition, which is characterized by trigger points and palpable cord-like indurations, requires a positive clenching test (21). This patient exhibits neither focal trigger points nor exacerbation of tenderness upon 5-second isometric jaw clenching, which effectively rules out masseter myofascial pain. Radiation-induced osteonecrosis, a complication of high-dose radiotherapy, manifests with characteristic jawbone radiolucency (22). The absence of a radiation exposure history, combined with normal tomographic findings, precludes this diagnosis.

Myofascial pain syndrome (MPS) of the masticatory muscles is a disorder characterized by regional pain and the presence of myofascial trigger points (MTrPs)—hyperirritable nodules located within taut bands of skeletal muscle that are painful on compression and can elicit referred pain (23). MPS is often considered in the differential diagnosis of FBS. Although the present case features a lack of trauma or surgical history and unresponsiveness to physical therapy, both of which are consistent with MPS—palpation of the major masticatory muscles (masseter, temporalis, and medial and lateral pterygoids) with standardized pressure reveals no detectable MTrPs (24). These findings effectively rule out MPS as a diagnosis in this patient. Through systematic exclusion, this case meets diagnostic criteria for FBS without confounding comorbidities.

Currently, the hypothesis that heat and exercise lead to abnormal neurological function lacks direct experimental support. Macartney et al. (25) demonstrated that high ambient temperature, physical exertion, and inadequate fluid intake can impair cardiac vagal nerve function, although the precise mechanism remains unclear. Several studies propose potential mechanisms underlying this process: Chen et al. (26) report that increasing temperature from 15 °C to 20 °C enhances the opening of sodium and potassium ion channels, thereby facilitating the generation of ankylotonic neural discharges. Austerschmidt et al. (27) observe that at elevated temperatures (30–33 °C), optic nerve axons undergo temperature-dependent hyperpolarization, a phenomenon linked to altered potassium ion leakage. Furthermore, Nascimento et al. (28), using transcriptomic analysis, reveal that temperature influences muscle responsiveness by modulating insulin sensitivity—a process potentially associated with reduced GLUT4 translocation. Despite these fragmented lines of evidence, a definitive mechanistic understanding of exercise-induced neurological and motor dysfunction under high-temperature conditions remains elusive.

A therapeutic consensus for first bite syndrome (FBS) remains elusive, with current evidence limited to case reports that demonstrate conflicting outcomes (29). Management strategies diverge by etiology: tumor resection consistently resolves malignancy-associated FBS, while idiopathic or traumatic cases require localized interventions (30). Non-invasive approaches include physiotherapy to mitigate neuromuscular hypertonicity and abnormal discharges, though their efficacy is variable (31). Pharmacological infiltration with gents such as carbamazepine or gabapentin may palliate symptoms but lacks a predictable response (32). Notably, botulinum toxin injection represents the most promising neurolytic intervention. It acts by blocking acetylcholine to paralyze myoepithelial cells, yet it faces challenges in standardizing dosage optimization and managing side effect profiles (33).

In our patient, initial physiotherapy failed to achieve significant improvement. Subsequent discussion of botulinum therapy was deferred due to patient preference, likely influenced by the episodic nature of symptoms (triggered only by post-exertional cold exposure) and successful behavioral adaptation through dietary modification.

During the peer Interactive -review process, the reviewers appropriately emphasized the importance of comprehensive neurological assessment and brain imaging to exclude central or neuropathic pathologies. Following detailed consultation, the patient declined further neurological workup, including brain MRI. Considering the distinctive and reproducible clinical features—highly specific triggering conditions, absence of motor or sensory deficits, and a long-standing stable course without progression—the authors maintain that idiopathic first-bite syndrome remains the most plausible diagnosis. Nevertheless, the inability to perform confirmatory neuroimaging or formal neurological testing represents a limitation of this case report. It is recommended that future evaluations of atypical FBS include enhanced brain MRI and systematic neurological examination as part of the standard diagnostic protocol.

This rare presentation has notable limitations. Reproducible electrophysiological capture of FBS episodes proved unfeasible due to the unpredictability of symptoms and because exercise-induced facial sweat compromised non-invasive EMG electrode adhesion. During the initial diagnostic stage, a systematic palpation examination for myofascial trigger points in the masticatory muscles was not performed. Following the reviewers' suggestion, additional palpation was conducted using standardized procedures, which revealed no detectable trigger points or tenderness in the major masticatory muscles, thereby effectively ruling out MPS as a likely diagnosis. The patient's declination of invasive procedures precluded direct confirmation of trigeminal V3 involvement through targeted neuromonitoring. Finally, the absence of longitudinal surveillance leaves potential occult pathology unruled out. Future follow-up will seek to address these constraints through extended observation.

## 5 Conclusion

We present a rare case of primary temperature-dependent first bite syndrome (FBS), demonstrating its specific provocation by post-exertional cold food intake in high ambient temperatures with neuroanatomically mapped symptomatic regions. This report provides novel mechanistic insights into FBS etiology by establishing the triad of thermal, metabolic, and mechanical triggers in idiopathic presentations.

## Data Availability

The original contributions presented in the study are included in the article/Supplementary Material, further inquiries can be directed to the corresponding authors.
